# The Role of *COL6A3* in Tumorigenesis, Metastasis, Diagnosis, and Disease Management

**DOI:** 10.3390/cancers17213449

**Published:** 2025-10-28

**Authors:** Joshua J. Lingo, Maggie M. Balas, Philipp E. Scherer, Jason C. Klein

**Affiliations:** 1 Department of Dermatology, University of Colorado Anschutz, Aurora, CO 80045, USA; 2 Touchstone Diabetes Center, University of Texas Southwestern Medical Center, Dallas, TX 75390, USA; 3 Department of Cell Biology, University of Texas Southwestern Medical Center, Dallas, TX 75390, USA

**Keywords:** collagen, *COL6A3*, endotrophin, tumorigenesis, metastasis, biomarker

## Abstract

**Simple Summary:**

Collagens are the most abundant proteins expressed in the human body and are a vital component of the extracellular matrix (ECM). Originally the ECM was thought to provide structural support to tissues. Over the past few decades, the ECM has also been implicated in cell signaling, tumor growth, and therapy resistance. Understanding how collagens, a family of proteins that comprise the majority of the ECM, regulate these signals is vital to combating their role in tumor progression. In this review, we discuss the role of *COL6A3*, the alpha 3 chain of type VI collagen, in the progression, spread, management, and prognosis of malignant disease.

**Abstract:**

Collagens comprise a large, diverse family of proteins that are abundantly expressed throughout most tissues. As a main component of the extracellular matrix (ECM), it is becoming increasingly appreciated how vital collagens are to tumor development, progression, and metastasis. *COL6A3*, which encodes the alpha 3 chain of type VI collagen, is a unique member of the collagen family that encodes a C-terminal peptide with powerful signaling capabilities, named endotrophin (ETP). Expression of *COL6A3* is required for the survival, migration, and invasion of many cancer cell lines, including breast, bladder, liver, and colorectal cancers. ETP, which was originally discovered to be enriched in the adipocytes of mammary tumors, is a powerful oncopeptide that can alter signaling of tumor and stromal cells. ETP has greater signaling potential than the parental *COL6A3* as it can induce EMT and promote chemoresistance, metastasis, and stemness in an TGF-β-like manner. ETP can also function independently of TGF-β to recruit endothelial cells and macrophages. In this review, we examine the molecular implications of *COL6A3* and ETP expression and their effects on tumor growth, metastasis, and therapeutic response. Finally, we speculate on the potential of serum ETP as a prognostic biomarker in both carcinomas and sarcomas. In summary, *COL6A3* and ETP are powerful drivers of tumor growth that have potential as noninvasive diagnostic and prognostic tools for the clinical management of cancer.

## 1. Introduction to Collagen Biology

The extracellular matrix (ECM) is a dynamic structure that provides physical support to tissues and tumors alike. The complexity of the ECM is largely attributed to the diversity of the proteins that comprise it, namely glycosaminoglycans, elastin, laminin, fibronectin, and collagen [1]. Remodeling of the ECM is critical for the genesis and metastasis of many cancers [2]. Understanding the individual contributions of the proteins that comprise the ECM is critical to understanding and preventing the dissemination of the primary tumor to distant sites. In this review, we focus on the role of type VI collagens, specifically *COL6A3*, in tumor development, progression, and metastasis. We conclude with commentary on the prognostic utility of *COL6A3* and a C-terminal cleavage product in predicting patient outcomes and therapy response.

### 1.1. Collagen, a Highly Diverse Family of Proteins

The tumor microenvironment is comprised of tumor, stromal, and immune cells as well as the ECM, which supports these cells within the tissue. The ECM has been implicated in many tumor-promoting processes such as tumorigenesis, therapy resistance, and metastasis [1]. The ECM is a dynamic structure comprised of many proteins but is predominantly composed of collagen. Collagen is one of the most abundant proteins in mammalian tissues and consists of 28 subtypes distributed across 8 families [3,4]. Disruption of collagen homeostasis has been shown to promote tumorigenesis in many settings [5].

Each family and subtype of collagen (summarized in Table 1) has unique roles within the ECM. Fibrillar collagens, which account for 90% of all collagens, form tightly packed fibrils that comprise the matrix in skin, bone, and tendons [6]. All other supramolecular families of collagens are termed “non-fibrillar” collagens, which are critical for maintaining tissue stiffness, anchoring cells to the ECM, and activating intracellular signaling pathways [7]. Fibril-associated collagens with interrupted triple helices (FACIT) are less abundant than fibrillar collagens and are critical for stabilizing and organizing the ECM [8]. Type IV collagens integrate laminins and other matrix proteins to form the highly organized basement membrane. Long-chain collagens anchor structures to the basement membrane and provide structural support whereas short-chain collagens bridge molecules together and support angiogenesis [9]. Multiplexin collagens maintain the integrity of the ECM [10]. Membrane-associated collagens with interrupted triple helices (MACITs) interact with extracellular molecules and aid signal transduction [3,11]. The last family of collagens, and the focus of this review, are filamentous, type VI collagens, which form beaded microfibrils that maintain tissue elasticity, regulate cell signaling, and control growth factor bioavailability [3,12].

### 1.2. Expression and Assembly of Type VI Collagen

To understand the role that filamentous type VI collagens play in tumor progression, we must first understand how each member of this family comes together to form beaded microfibrils in the ECM. Originally, three loci were discovered that encoded type VI collagens: *COL6A1*, *COL6A2*, and *COL6A3* [15]. *COL6A1* and *COL6A2* encode collagens α1(VI) and α2(VI), respectively, which are relatively small type VI collagens, around 130–150 kDa [15]. Structurally, α1(VI) and α2(VI) are very similar, with globular N- and C-terminal domains comprised of multiple von Willebrand factor type A (vWF-A) motifs linked by a triple helix collagenous domain. However, α3(VI) is much larger (250–300 kDa), with nine additional N-terminal vWF A motifs and three distinctive C-terminal domains [16,17,18,19,20]. The C-terminal end of α3(VI) is characterized by a unique domain (C3), a fibronectin type III domain (C4), and a Kunitz-like domain with powerful signaling capabilities (C5). This C5 domain is referred to as endotrophin (ETP) [15,17,20,21,22]. Intriguingly, deficiencies in α1(VI) abrogates the secretion of α2(VI) and α3(VI), highlighting the importance of the heterotrimer in the formation of mature type VI collagens [23].

In recent years, three new isoforms of type VI collagen were discovered: *COL6A4*, *COL6A5*, and *COL6A6*. In humans, *COL6A4* is disrupted by a chromosomal break that resulted in the formation of two pseudogenes that are not expressed [13,14]. However, *COL6A4* is expressed in mice as α4(VI), although its contribution to the formation of mature type VI collagens is not well-understood. *COL6A5* and *COL6A6*, which encode collagens α5(VI) and α6(VI), are structurally very similar to α3(VI) [13,24]. The collagenous domains of α4(VI), α5(VI), and α6(VI) are identical in size to the domain encoded within *COL6A3*, suggesting that they may be able to substitute for α3(VI) in heterotrimers with α1(VI) and α2(VI) [24]. Additionally, mice lacking α1(VI) are unable to express α4(VI), α5(VI), or α6(VI). However, there is currently no direct evidence to support the oligomerization of new collagen VI alpha chains with 1(VI) or 2(VI).

Collagen assembly is a complex process involving protein translation, aggregation and folding, and extracellular enzymatic digestion. Original studies employing electron microscopy to visualize collagen synthesis over time found that type VI collagens are synthesized from monomers of α1(VI), α2(VI), and α3(VI), which are assembled in a 1:1:1 manner [15,25,26,27]. In the endoplasmic reticulum, the three alpha chains form triple helix monomers. These monomers then are staggered, organized in an anti-parallel manner, and dimerize via disulfide bonding. Finally, dimers are joined through additional disulfide bonds to form tetramers that are then secreted to the ECM through the vesicular network. After secretion, tetramers are connected to one another via non-covalent bonds between neighboring N-terminal globular domains, forming beaded microfibrils [26,27,28,29,30]. Disease-driving mutations observed within protein-coding regions of the *COL6* loci are often restricted to trimeric-coiled coil and triple-helical regions that are critical for the assembly of heterotrimers or microfibrils, respectively [31].

Full maturation of type VI collagens involves the enzymatic cleavage of C-terminal domains, which results in either the production of a C2–C5 fragment, or the C5 motif alone, which encodes ETP [15,25,27,31,32,33]. Importantly, the presence of the C5 fragment is not required for the assembly of the mature collagen VI molecule [34]. Recently, matrix metalloprotease 14 (MMP14) [35] and bone morphogenetic protein 1 (BMP-1) [32] were shown to cleave at the C terminus of α3(VI), producing ETP. It is likely that other proteases with activity at this site exist and may represent potentially actionable therapeutic targets in ETP-rich tumors.

## 2. *COL6A3* in Cell Signaling and Tumor Progression

Much of what we know about type VI collagens in promoting malignant disease comes from breast cancer, where it was originally characterized [36]. The main source of type VI collagen in the human body is adipose tissue [37], which comprises a large portion of the breast tumor microenvironment [38]. Adipocytes, amongst other stromal cells, are thought to aid tumorigenesis and metastasis through a variety of secretory factors [39] including α3(VI). Early studies using mice genetically ablated for *COL6A1*, which resulted in a functional deficiency in type VI collagen, showed that *COL6A1* was critical for the growth of allografted breast cancer cells [21]. While there is a tumor-promoting role for type VI collagen when expressed within the stromal compartment, tumor-specific expression can also support tumor progression. Knockdown of endogenous *COL6A3* suppresses the proliferation of bladder [40] and colorectal [41] cancer cell lines in vitro. In colorectal cancer, Cas9-mediated knockout of *COL6A3* reduced cell proliferation, migration, and induced early apoptosis [41]. However, the mechanisms by which these phenomena occur in colorectal cancer are currently unknown.

**Figure 1:**
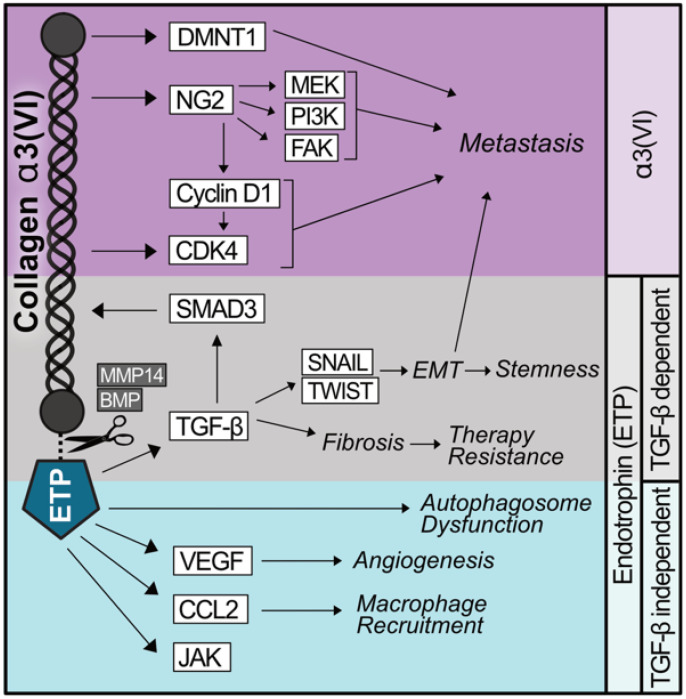
Shared and diverging signaling capabilities of α3(VI) collagen and ETP. α3(VI) promotes the expression of the oncoproteins CDK4/6 and DNMT1, which promote metastasis. α3(VI) binds NG2 which stabilizes Cyclin D1, activates CDK4/6, and independently activates MEK, PI3K, and FAK, contributing to α3(VI) driving metastasis. The C-terminal domain of α3(VI) encodes a peptide, named endotrophin (ETP), which is produced as a result of the proteolytic cleavage of α3(VI) by MMP14 or BMP. ETP prompts an epithelial–mesenchymal transition (EMT)-like phenotype in a TGF-β-dependent manner, promoting therapy resistance, stemness, and metastasis. ETP also functions to recruit macrophages and endothelial cells as well as augment autophagy independently of TGF-β.

Recently, type VI collagen has been shown to promote the migration and invasion of the SKOV3 ovarian cancer cell line when expressed by ovarian cancer stromal progenitor cells (OSCPCs) grown in co-culture [42,43]. Knockdown of *COL6A3* using shRNA reduces spheroid formation; however, the addition of recombinant type VI collagen to co-culture systems increases the spheroid formation and invasiveness of SKOV3 cells. Intraperitoneal injection of SKOV3 cells with OCSPCs prompts widespread metastases in the peritoneum that are blunted in mice co-injected with *COL6A3* knockdown OCSPCs [42]. Conversely, the overexpression of α3(VI) greatly increases the peritoneal dissemination and lung colonization of SKOV3 cells when injected intraperitoneally or intravenously, respectively [43]. Mechanistically, *COL6A3* overexpression in SKOV3 cells increases the expression of three oncoproteins, cyclin-dependent kinases 4 and 6 (CDK4/6) and DNA methyltransferase 1 (DNMT1) (Figure 1). Treatment of ES2 cells with the CDK4/6 inhibitor, ribocliclib, significantly reduces the invasiveness of spheroids. Ribociclib reduces α3(VI) expression and is effective in reducing the invasiveness of chemotherapy-resistant ES2 spheroids, suggesting that it may be effective in combating acquired therapy resistance [42]. It is currently unknown whether α3(VI), ETP, or both promote the expression of CDK4/6 and DNMT1.

In bladder cancer, we have a better mechanistic understanding, where *COL6A3* activates the transforming growth factor β (TGF-β) pathway. After transient silencing of *COL6A3* using gene-specific siRNAs, bladder cancer cell lines expressed less TGF-β and exhibited less SMAD2/3 activity, indicated by their hypophosphorylation [40]. These data suggest that α3(VI) can promote tumor proliferation, migration, and confer resistance to apoptosis. Since the loss of α3(VI) significantly alters the assembly of mature collagen VI [25,27,28], more nuanced studies to dissect the individual roles of α3(VI), collagen VI, and global matrix remodeling are needed. Interestingly, the analysis of pan-cancer TCGA data showed that *COL6A3* as well as *COL6A1* and *COL6A2* are expressed most highly in the C6 immune subtype, which is TGF-β dominant [44]. Exogenous TGF-β administration in mouse dermal fibroblasts induces a rapid upregulation of both *COL6A1* and *COL6A3* in a SMAD3-dependent manner, suggesting that *COL6A3* and TGF-β may be linked through reciprocal signaling pathways [45]. Activation of TGF-β is critical for maintaining tissue homeostasis but is often dysregulated in cancers, promoting tumor development and metastasis through supporting the epithelial-to-mesenchymal transition (EMT) [21,46]. However, to fully understand the relationship between *COL6A3* and TGF-β, we must also discuss the C5 domain of *COL6A3*, ETP.

## 3. Endotrophin, a Powerful Signaling Peptide Encoded by *COL6A3*

### 3.1. ETP and Known Signaling Partners

While type VI collagens directly regulate mechanosensation and signal transduction, ETP possesses signaling capabilities not shared by the parent α3(VI). In 2005, the C-terminal end of α3(VI) was observed to be highly upregulated in malignant breast tissue compared with healthy controls, and this increase persisted as the disease progressed [36]. Radiographic imaging using radiolabeled antibodies specific to ETP revealed that ETP accumulates rapidly at the tumor site and remains elevated, inciting speculation that the protein is expressed at higher rates or becomes more stable during malignant disease. The same group demonstrated that type VI collagen stabilizes Cyclin D1, a process that can be reversed by blocking the type VI collagen receptor, NG2 (also known as chondroitin sulfate proteoglycan 1 or CSPG) [36]. Signaling through NG2 by type VI collagens is a cooperative effort requiring local integrins and receptor tyrosine kinases that support the activation of kinases like focal adhesion kinase (FAK), mitogen-associated protein kinase (MAPK), and phosphatidylinositol 3-kinase (PI3-K) [47]. Importantly, these kinases play prominent roles in tumor metastasis [48].

### 3.2. TGF-β-Dependent Signaling by ETP

ETP is hypothesized to interact with other receptors including different integrins [49] and the anthrax toxin receptors 1 and 2 (ANTRX1 and ANTRX2) [50]. In 2004, ANTRX1 was identified as an ETP binding partner through a yeast-two-hybrid screen [51]. Using the extracellular domain of ANTRX1 as bait, the St. Croix group identified the C terminus of α3(VI) as an ANTRX1-interacting protein. In 2016, ANTRX2 was found to bind collagen VI coated plates but was unable to bind plates coated with collagens I or IV. Additionally, plating cells on collagen VI coated plates increased ANTRX2 phosphorylation, resulting in the internalization and degradation of collagen VI. These reports did not identify to which region of collagen VI that ANTRX2 binds; however, the collagen VI employed for this study only included the triple helical domain. Therefore, it is unlikely that any signaling through ANTRX2 by α3(VI) is not due to interactions between ANTRX2 and ETP.

Despite original speculation that ETP interacts with ANTRX1 and ANTRX2, a more recent report was unable to reproduce any interaction between ETP and either protein [52]. Using surface plasmon resonance and cell-based immunofluorescence, ETP was found to not interact with ANTRX1 or ANTRX2. A known binding partner of ANTRX1 and ANTRX2, named protected antigen, was used as a positive control and exhibited robust binding and colocalization with both receptors [52]. In this most recent report, the authors speculated that the yeast-two-hybrid system may be insufficient to study extracellular binding events in mammalian cells. Specifically, the investigators suggest that the reducing nature of the nucleus, where the screen takes place, may hinder key structural modifications and introduce bias when studying protein–protein interactions that occur in the extracellular space. Advancements in sequencing and imaging technologies will likely be key modalities to discover putative ETP binding partners. Recently, spatial transcriptomics paired with 3D ECM imaging highlighted an interaction between *COL6A3* and Integrin β1 (*ITGB1)* [49]. Experimentally it has not been validated as to which region of α3(VI) may interact with other integrins; however, advancements in predicting protein–protein interactions will likely identify other potential ETP binding partners.

While the search for ETP receptors is ongoing, understanding the molecular consequences of ETP expression may provide clues into key interacting partners. The first study to overexpress ETP under the mouse mammary tumor virus (MMTV) promoter found that ETP modestly accelerated the growth of primary tumor induced by the expression of polyoma middle T antigen (PyMT) [21]. Overexpression of ETP alone was sufficient to increase the number and rate of lung metastases [21]. Original histological analyses of ETP-overexpressing murine mammary tumors showed that ETP promotes tumor fibrosis, angiogenesis, and inflammation, all of which can promote metastasis [21,53] (summarized in Figure 1). ETP promotes metastasis by inducing EMT-like phenotypes in the primary tumor, reducing the expression of E-cadherin, and increasing the expression of vimentin. These effects could be ameliorated in vivo by the systemic administration of a TGF-β neutralizing antibody, which suppressed the EMT phenotypes, tumor metastasis, and primary tumor growth.

Overexpression of ETP in primary mammary tumors promotes the transcription of *Tgfb1*, although the mechanism by which this occurs is unknown [21] (Figure 1). Luciferase reporter assays employing SMAD binding elements demonstrated that ETP increases SMAD activity, which can be enhanced or blunted through the administration of recombinant TGF-β or an anti-TGF-β antibody, respectively. Nevertheless, ETP supports the dissemination of tumor cells from the primary site through the TGF-β-dependent induction of EMT [15,21]. Treatment of human breast cancer cell lines with recombinant ETP enhanced the transcription of *Twist* and *Snail*, two hallmark EMT transcription factors critical for promoting metastasis [54]. MCF-7 cells overexpressing ETP grafted into nude mice expressed more *Twist* and *Snail*, exhibited reduced E-cadherin expression, and increased the number of intratumoral, Mac2+ macrophages when compared with the control xenografts. Neutralizing antibodies against ETP attenuated the growth of MDA-MB-231 xenografts and improved the efficacy of cisplatin treatment against nascent and established tumors [22].

TGF-β is a pleiotropic signaling molecule. Currently, the ability of ETP to promote other TGF-β related phenotypes, like stemness and immune evasion, has been unexplored. However, microarray profiling from PyMT/ETP tumors uncovered an upregulation of pathways related to the cell cycle, pluripotency, Janus kinase (JAK) signaling, and immune checkpoint proteins [21], suggesting that the ETP/TGF-β axis may also promote tumor progression in an EMT-independent manner. Future studies aimed at discerning the differential abilities of ETP and TGF-β to signal through multiple signaling pathways will be informative in developing targeted therapies for ETP-rich cancers.

### 3.3. TGF-β-Independent Signaling by ETP

Much of what we know about ETP signaling in cancer can be traced back to its function in normal adipocytes. ETP is rapidly degraded by adipocytes; however, in settings of obesity, endosomal ETP escapes the lysosome and accumulates in the cytosol [55]. Treating primary adipocytes with recombinant ETP promotes the association of coat protein complex II (COPII) vesicles with autophagy-related gene 7 (ATG7), leading to an increase in autophagosome formation (Figure 1). Intracellular accumulation of ETP can induce cell death in primary adipocytes. The mechanism by which this would alter metabolism in cancers that have developed resistance to apoptosis is unknown, although there are no existing data to suggest whether ETP augments the vesicular trafficking network in malignant settings. If so, ETP may indicate sensitivity to autophagy-modulating therapies, which can be particularly useful in combination with chemoresistant cancers [56]. TGF-β has been shown to activate autophagy as a tumor protective mechanism in hepatocellular carcinoma (HCC) cell lines, so it is unlikely that ETP signals through TGF-β to promote autophagic dysfunction [57,58].

In addition to signaling through the TGF-β pathway, ETP is a potent chemokine and can induce angiogenesis in a TGF-β-independent manner. Exogenous expression of ETP within the PyMT murine mammary tumor model promotes the transcription of *Hif1a* and significantly increases the number and functionality of blood vessels in the primary tumor [21]. Overexpression of ETP reduces pimonidazole-HCl labeling of hypoxic tumor cells by over 50%. Matrigel invasion assays in vivo demonstrated that ETP promotes the migration of the endothelial cell line, MS-1. ETP expression slightly increased the expression of *Vegfr2*, *Vegfa*, and *Pecam1* as well as the blood vessel area and density. In vitro migration assays employing human umbilical vein endothelial cells (HUVECs) showed that the exogenous administration of ETP stimulated tube formation and vascular reorganization [21,22] (Figure 1). Culturing endothelial cells in conditioned media from ETP-overexpressing HEK293T cells also increased motility and vessel formation [21]. Importantly, recombinant ETP increased the motility and migratory capabilities of HUVECs in a dose-dependent manner [22].

ETP can also recruit primary macrophages, even toward target cells that lack the endogenous expression of type VI collagen [21] (Figure 1). In obesity, where *COL6A3* expression is upregulated, adipocyte-adjacent macrophages adopt an M2-like, immunosuppressive phenotype. Understanding the contribution of ETP to macrophage polarization may be critical to improving therapy response in macrophage-rich cancers like soft tissue sarcomas [59]. Similar to its role in endothelial cells, ETP serves as a chemokine for macrophages, enhancing migration when used as bait in transwell migration assays [21]. Antibodies targeting TGF-β do not ameliorate any stromal motility, indicating that ETP does not require TGF-β to function as a chemokine [21]. Thus, ETP has unique TGF-β-independent functions as a chemokine that are not shared by type α3(VI) (as summarized in Figure 1).

Oncogenes that drive matrix remodeling, particularly those that do so through the regulation of α3(VI), likely alter ETP expression and function as a result. In undifferentiated pleomorphic sarcoma (UPS), the oncogenic driver yes-associated protein 1 (*YAP1*) was found to promote matrix remodeling, specifically through increasing the deposition of α3(VI) into the extracellular matrix [60]. Tumors with elevated *YAP1* and α3(VI) also exhibited dysfunctional CD8+ T cells, characterized by the increased expression of the exhaustion markers programmed cell death protein 1 (PD-1) and T-cell immunoglobulin and mucin-domain containing-3 (TIM-3). Suppression of *YAP1* is sufficient to improve T-cell killing of UPS cells in co-culture assays. Furthermore, loss of *Yap1* in the *Kras*^G12D/+^; *Trp53*^fl/fl^ mouse model of skeletal-muscle derived UPS sensitized tumors to immune checkpoint blockades targeting PD-1 [60]. Interestingly, loss of *Yap1* did not alter macrophage polarization or infiltration into UPS tumors. It is currently unknown whether *YAP1* expression augments the expression or bioavailability of ETP. These reports indicate that α3(VI) and ETP have shared and distinct roles in promoting tumor proliferation, immune dysfunction, and metastasis. Dissecting the individual signaling capacities of ETP and type VI collagen will be critical in the development of novel diagnostic technologies and therapeutic interventions.

## 4. *COL6A3* in Predicting Therapy Response and Resistance

### 4.1. Relationship Between α3(VI) and Anti-Neoplastic Therapy Response

In 2003, Serial Analysis of Gene Expression (SAGE) sequencing of ovarian cancer cells with acquired resistance to cisplatin showed a 12-fold increase in *COL6A3* mRNA in therapy-resistant cells compared with the parental lines [61]. Gene expression analysis of the ovarian cancer cell line A2780 after dose escalation and the development of acquired resistance to oxaliplatin and cisplatin revealed a 62-fold increase in *COL6A3* transcript levels compared with therapy-sensitive clones, supporting the original reports [62]. More recently, the Persano group demonstrated a role for *COL6A3* in glioblastoma multiforme (GBM), where it is highly upregulated compared with normal brain and benign gliomas [63]. α3(VI) exhibits a gradient-like staining pattern with more intense staining near the center of the GBM, which is known to be enriched for stem-like cells [63,64]. Indeed, α3(VI) immunoreactivity colocalizes with Nestin expression, which marks neural stem cells [63,65]. α3(VI) expression prevents differentiation in Nestin+ GMB stem-like cells and blocks mitogen-induced differentiation using fetal bovine serum. Silencing of *COL6A3* in GBM cells suggested a role for α3(VI) in DNA damage response pathways, which was validated via Western blotting, showing reduced yH2aX in α3(VI)-depleted cells. Indeed, interfering with endogenous α3(VI) sensitizes cells to temozolomide, a DNA alkylating agent, increasing both DNA damage and cell death [63]. Whether *COL6A3* confers resistance to different chemotherapeutic agents through similar mechanisms is currently unknown.

α3(VI) and matrix remodeling have been identified as critical regulators of chemoresistance in many cancers; however, less is known about sensitivity to targeted therapies. Using the NCI-60 cell line expression data, *COL6A3* was correlated to therapy response. *COL6A3* expression inversely correlated with responses to MEK inhibitors, histone deacetylase (HDAC) inhibitors, and nucleoside analogs [44]. However, *COL6A3* expression correlated with favorable responses to many drug classes. These include, but are not limited to, inhibitors of mammalian target of rapamycin (mTOR), PI3-K, topoisomerase II, pan-receptor tyrosine kinases, and the hedgehog pathway. To our knowledge, there are no reports validating the response to any of these drug classes in preclinical models perturbing *COL6A3.* Nevertheless, these drug classes represent potentially exciting, targeted therapies or combination therapies to combat tumors that express high levels of *COL6A3* like breast cancer, UPS, and pleomorphic dermal sarcoma (PDS).

### 4.2. Targeting ETP to Improve Chemotherapy Response

While α3(VI) has been posited as a potential regulator of chemoresistance, ETP may also play a direct role. Much like in ovarian cancer, *COL6A3* is highly upregulated in cisplatin-resistant mammary tumors [66]. Development of mammary tumors in PyMT mice deficient in type VI collagen increases tumor sensitivity to cisplatin treatment. Sensitivity to cisplatin in type VI collagen knockout PyMT mice is lost upon the systemic administration of ETP. In agreement, overexpression of ETP on the PyMT background confers resistance to both high and low doses of cisplatin [66]. Tumors with acquired resistance to cisplatin or the overexpression of ETP underwent EMT, characterized by reduced E-cadherin and increased Vimentin. Neutralizing anti-ETP antibodies slow tumor growth, reduce lung metastases, prevent EMT, and mitigate cisplatin resistance in tumor-bearing PyMT mice. Ongoing work is being conducted to determine the utility of therapeutically targeting ETP; however, a recently developed ETP knockout (KO) mouse showed no physiological deficiencies compared with wild-type mice until challenged with fibroinflammatory stressors [34].

Outside of neutralizing monoclonal antibodies, there are no specific inhibitors of ETP that exist; however, data suggest that peroxisome proliferator-activated receptor gamma (PPAR) agonists, such as thiazolidinediones (TZDs), suppress the protumoral effects of ETP. TZDs are relatively safe therapeutics for patients with type 2 diabetes mellitus; however, specific TZDs carry higher risks of cardiovascular complications, limiting their use in patients with comorbid cardiovascular conditions [67]. Treating PyMT tumor-bearing mice with TZD has little effect on tumor proliferation at the baseline; however, it has powerful anti-proliferative effects against tumors overexpressing *ETP* [66] and reduces the severity of adverse effects that patients experience in response to insulin-sensitizing PPAR agonists [68]. Patients diagnosed with type 2 diabetes mellitus that are treated with TZDs are also more likely to respond to therapy if they have higher levels of serum ETP, highlighting its potential to predict therapy response in some settings [68]. Whether serum ETP or α3(VI) can be used as a biomarker of anti-neoplastic therapy response has yet to be evaluated in a human cohort.

## 5. The Diagnostic and Prognostic Utility of *COL6A3* and ETP

### 5.1. The Prognostic Capabilities of COL6A3

Within the family of type VI collagens, the high expression of *COL6A3* and ETP has been implicated in the progression of cancers, adverse renal events, metabolic syndromes, and liver disease [15,44,69,70,71,72]. Recent pan-cancer analysis of bulk RNA sequencing data from The Cancer Genome Atlas (TCGA) demonstrated that *COL6A3* is transcriptionally upregulated in 13 out of 18 cancer types, relative to their respective normal tissues. Additionally, the same study found that *COL6A3* expression was significantly inversely correlated with patient survival in 11 out of 18 cancer types [44] (summarized in Table 2). Both mRNA and protein levels of *COL6A3* are highly upregulated in many cancers including those of the breast, ovaries, colon, pancreas, and liver [15,42,73]. Though there are many mechanisms by which protein expression is altered throughout tumor progression, methyl-sensitive cut counting in glioblastoma revealed that CpG islands within *COL6A3* are significantly demethylated, implicating epigenetic modifiers as a potential regulator of α3(VI) expression [74]. Somewhat paradoxically, *COL6A3* mRNA expression is suppressed as prostate cancers become more aggressive, highlighting a context-dependent nature of how type VI collagens augment tumor biology [75]. It could be the case that tissue-specific microenvironments differentially remodel α3(VI) to allow tumor progression, although this has not been tested. However, ETP is highly upregulated in the periprostatic adipose tissue of aggressive prostate tumors due to the degradation of α3(VI) [76]. These studies highlight the importance of determining the contribution of the tumor and stromal compartments when studying *COL6A3* and ETP.

Understanding the specific matrix composition within an organ of interest will be critical in understanding the role α3(VI) plays in metastasis across cancer subtypes. For example, in UPS, collagen I antagonizes collagen VI, functioning as a tumor suppressor and promoting T-cell function [60]. Understanding the relationship within supramolecular collagen families will likely improve our understanding of tissue-specific matrix remodeling. Nevertheless, most available reports implicate high *COL6A3* expression as a prognostic biomarker of poor patient outcomes [15,44]. Advancements in matrix modeling, like decellularized matrix scaffolds derived from breast tumors [77], will likely be vital in studying the specific role of α3(VI) in promoting tissue-specific metastasis.

*COL6A3* expression has been reported as a biomarker of disease progression and recurrence in cancers of the breast, colon, and liver. In colorectal cancers, high expression of *COL6A3* at the mRNA or protein level predicts poor prognosis, advanced clinical staging, and disease recurrence [78]. Proteomic studies on colonic fibroblasts and cultured cancer cells have shown that *COL6A3* is enriched in the stroma of colon cancers, which was validated at the transcript and protein level [79]. Additionally, this group reported elevated α3(VI) protein in the plasma of colorectal cancer patients compared with healthy controls, suggesting that it may be a useful biomarker to aid in the diagnosis of colon cancers [79]. Analysis of the TCGA datasets demonstrated minor associations with the expression of *COL6A3* and poor survival in colon and liver cancers; however, *COL6A3* predicted 2-fold or higher mortality in renal cancers and uveal melanoma [44]. While the expression of *COL6A3* has been suggested as a potential biomarker, mutations within the coding regions of *COL6A3* may also have a profound impact on prognosis. In hepatocellular carcinoma, patients with mutated *COL6A3* exhibited a 3.5-fold higher mortality rate than those with wild-type *COL6A3* [80]. However, in colorectal cancer, *COL6A3* is part of a five-gene-signature in which mutations in at least one gene are significantly associated with enhanced overall survival, irrespective of tumor-node-metastasis staging [81]. These data indicate a critical role for *COL6A3* in the development and progression of multiple cancers.

Particularly in the case of rare cancers, there are few validated biomarkers that can diagnose, prognose, and monitor disease status. *COL6A3* may be a useful biomarker in sarcomas, where high expression is associated with poor patient survival in UPS [70]. Recently, the first single-cell RNA sequencing of patient atypical fibroxanthoma (AFX) and PDS, two dermal-based soft tissue sarcomas, revealed that *COL6A3* is expressed by both tumor and stromal cells, and tumor expression is upregulated in the more aggressive PDS than AFX [70]. It could be that *COL6A3* is critical for the progression of rare tumors, although survival analyses of specific sarcoma subtypes are often difficult due to limited cohort sizes. Despite this limitation, *COL6A3* may have profound utility in the diagnosis, prognosis, and management of sarcomas as it does in epithelial cancers.

### 5.2. The Prognostic Utility of Endotrophin

ETP, the C-terminal cleavage product of α3(VI), has also been investigated as a serum biomarker in several cancers. ETP was first identified as being enriched in the serum of breast cancer patients [22]. Additionally, serum ETP is elevated in patients with liver cirrhosis and HCC as detected by the PRO-C6 ELISA, which is commercially available from Nordic Bioscience (NordicPRO-C6™). Higher levels of serum ETP correlate with worse overall and progression-free survival in HCC and are markedly more prognostic if measured alongside alpha-fetoprotein (AFP), a known biomarker of HCC survival [71,82]. Serum levels of AFP or ETP alone are sensitive enough (65.1% versus 79.8%, respectively) to accurately detect the presence of HCC compared with non-tumor bearing patients with cirrhosis. However, AFP is more specific than ETP (80.2% versus 50%, respectively). Currently, we are unaware of any reports indicating the utility of serum ETP as a biomarker of survival of any other cancers. Given the role of ETP in promoting EMT, there is also rationale to pursue ETP as a biomarker of metastasis, therapy resistance, or recurrence, particularly in ETP-driven cancers like breast and HCC. However, fibroinflammatory conditions like liver disease, chronic kidney disease, and metabolic disease are also associated with elevated serum ETP [83,84,85]. Consequently, the utility of ETP as a biomarker for survival in cancers is often confounded by comorbidities arising in patients with severe disease, limiting assay specificity. It is likely the case for many cancers, as it is for HCC, that ETP may hold prognostic value when assayed in concert with other biomarkers.

## 6. Conclusions

Collagens are among the most abundant proteins in the human body and comprise a large portion of the ECM. The ECM is a dynamic structure that is critical for providing mechanical support to tissues. During tumorigenesis, the ECM is remodeled, which represents a critical hurdle in understanding cancer progression, therapy response, and metastasis. *COL6A3* encodes non-fibrillar, type VI collagen, which possesses unique signaling characteristics at least in part due to ETP, a C-terminal cleavage product. α3(VI) and ETP can signal through TGF-β-dependent and -independent pathways, promoting EMT, cell stemness, therapy resistance, and the dissemination of the primary tumor to distant sites. ETP can also remodel the stromal compartment as it promotes angiogenesis in situ and recruits macrophages to the tumor site. Therapeutically, ETP can be targeted using neutralizing antibodies or indirectly with the treatment of PPARγ agonists such as TZD. Blocking ETP impedes many of the tumor-promoting consequences of α3(VI) and ETP expression. Finally, ETP may have significant utility as a serum biomarker for disease presence, progression, and recurrence. In summary, α3(VI) and ETP promote the progression of many cancers, can be therapeutically targeted, and may be valuable in the diagnosis and prognosis of ETP-driven cancers.

## Figures and Tables

**Table 1:** Collagen family classifications and protein coding loci.

Family		Type	Protein Coding Gene
Fibrillar		I	*COL1A1* and *COL1A2*
		II	*COL2A1*
		III	*COL3A1*
		V	*COL5A1*, *COL5A2*, and *COL5A3*
		XI	*COL11A1*, *COL11A2*, and *COL11A3*
		XXIV	*COL24A1*
		XXVII	*COL27A1*
Non-fibrillar	FACIT	IX	*COL9A2*, *COL9A2*, and *COL9A3*
		XII	*COL12A1*
		XIV	*COL14A1*
		XVI	*COL16A1*
		XIX	*COL19A1*
		XX	*COL20A1*
		XXI	*COL21A1*
		XXII	*COL22A1*
	Basement membrane	IV	*COL4A1*, *COL4A2*, *COL4A3*, *COL4A4*, *COL4A5*, and *COL4A6*
	Long-chain	VII	*COL7A1*
	Short-chain	VIII	*COL8A1*
		X	*COL10A1*
	Multiplexin	XV	*COL15A1*
		XVIII	*COL18A1*
	MACIT	XIII	*COL13A1*
		XVII	*COL17A1*
		XXIII	*COL23A1*
	Filamentous	VI	*COL6A1*, *COL6A2*, *COL6A3*, COL6A4 *, *COL6A5*, and *COL6A6*

* *COL6A4* is not expressed as a functional protein in humans [13,14].

**Table 2:** Prognostic significance of *COL6A3*, α3(VI), and ETP across cancer types.

Type	Biomarker	Correlation with Outcome	Ref.
Breast	*COL6A3*	Elevated mRNA predicts poor survival	[77]
	ETP	Serum ETP is elevated in cancer patients	[22]
Colorectal	*COL6A3*	mRNA predicts poor prognosis and recurrence	[78]
	α3(VI)	α3(VI) is upregulated in cancer stroma	[79]
Liver	*COL6A3*	Elevated mRNA predicts poor prognosis	[80]
		Mutations predict favorable outcomes	[81]
	ETP	Serum ETP is predictive tumor burden	[71]
Pancreatic	*COL6A3*	mRNA is elevated in malignant tissue	[73]
Ovarian	*COL6A3*	Elevated mRNA predicts poor survival	[42]
Prostate	*COL6A3*	mRNA decreases as tumor stage progress	[75]
Renal	*COL6A3*	Elevated mRNA predicts poor survival	[44]
Uveal Melanoma	*COL6A3*	Elevated mRNA predicts poor survival	[44]
UPS	*COL6A3*	Elevated mRNA predicts poor survival	[70]
AFX/PDS	*COL6A3*	mRNA elevated in more aggressive PDS	[70]

## Data Availability

No primary data were generated in this review.
